# Supplementary material for: Nicotine alters cellular activity and mRNA expression of patterns of Astrocytes
Source: PLoS One. 2025 Jun 20;20(6):e0325529. doi: 10.1371/journal.pone.0325529 (PMC12180639; doi:10.1371/journal.pone.0325529)
Supplement: Supplementary Fig 1 — (DOCX) [file pone.0325529.s001.docx]

**Supplement**


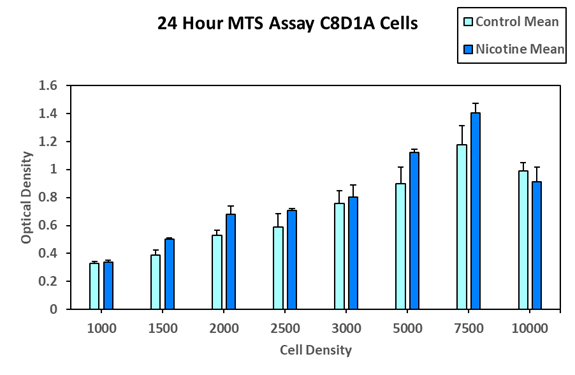

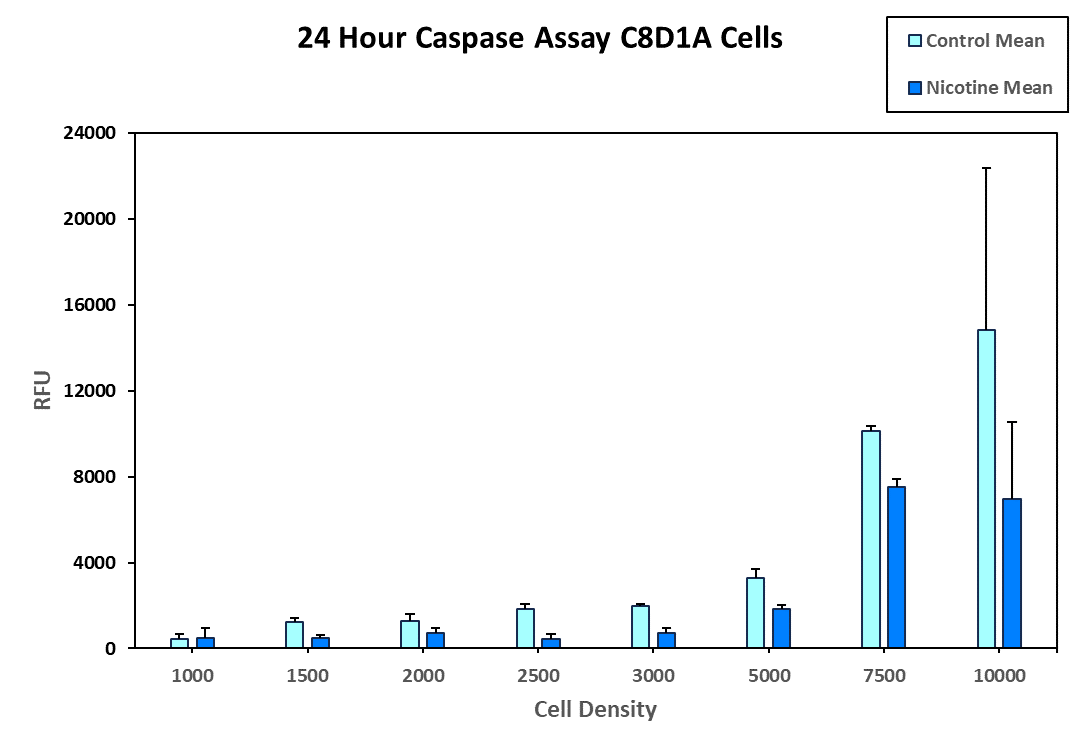


**Supplementary Figure 1:** Preliminary data used to establish an optimal cell density and determine the nicotine concentration levels of interest. Based on these cell viability assays, an optimal cell density of 2500 cells per well was determined.
