# Supplementary material for: Nicotine alters cellular activity and mRNA expression of patterns of Astrocytes
Source: PLoS One. 2025 Jun 20;20(6):e0325529. doi: 10.1371/journal.pone.0325529 (PMC12180639; doi:10.1371/journal.pone.0325529)
Supplement: Attachment — Submitted filename: Comments-PONE-D-24-55873.docx [file pone.0325529.s003.docx]

Comments:

The manuscript addresses an important topic. The study tests the effect of nicotine on murine astrocytes, focusing on cellular activity and mRNA expression. The results indicate that Nicotine impacts astrocyte activity and inflammatory signaling, suggesting its potential involvement in neuroinflammation. For better clarity the following revision is recommended:

1. The abstract as well as introduction should be modified to describe Tobacco/Nicotine consumption as a major global health concern/risk in general (in adults and in pregnancy), as nicotine teratological effect in astrocytic embryonic development was not specifically assessed in this paper. C8D1A cell line was used (Postnatal d8 astrocytic cerebellum).
2. The introduction section should be expanded to include:
a. Nicotine absorption, concentration in blood and brain, half-life (2 hours) etc. This would help understand the relevancy of selected doses and culture feeding regimen used in the study.
b. Summary of research exploring the effect of Nicotine on astrocytes should be expanded, include studies demonstrating the expression of nicotinic acetylcholine receptors (nAChRs) in astrocytes, and its downstream effects on astrocytic signaling pathways, morphological and functional changes etc.

Some recommended refs:

a. Aryal et al, Glia. 2021 Apr 14;69(8):2037–2053. doi: [10.1002/glia.24011](https://doi.org/10.1002/glia.24011)
b. Stellwagen et al 2019, Curr Opin Neurobiol 57: 179–185. [doi.org/10.1016/j.conb.2019.02.010](https://doi.org/10.1016/j.conb.2019.02.010).
c. Hernández-Morales et al, 201, Neuroscience 2014
3. Material and Methods:
a. Please include media change regimen (e.g. daily? Every other day? Etc) – might affect results interpretation (Acute exposure vs. chronic).
b. Include Nicotine source and Cat number.
4. Results:
a. MTS assay: The assay relies on cellular metabolic activity, which may not always directly correlate with cell number, if feasible please correlate with number of counted cells (e.g. neuclocounter/Hemcytometer, ICF Dapi count), if not applicable, address this issue in discussion section.
b. Figure 1. Where applicable please add statistical significance asterisks (e.g. in comparison with non-treated-NT arm (0ng/ml) at same time point). The Y axis should be modifies to represent Cell Viability (measured by OD).
c. The reduction in cell activity/cell number or metabolic activity in un-treated arm after 24 & 48 hours compared to 12&18hr should be explained.
d. Figure 2. – Where applicable statical significance (asterisks) compared to NT should be added, Y-axis (apoptosis – measured by RFU – add abbreviation (Relative Fluorescence Units).
e. The peak in mRNA expression (in both A1/A2 markers graphs) occurs after 48 hr (higher fold change – pls note difference in scaling), should be discussed/explained.

Discussion section:

1. The effect of Nicotine was assessed only in one murine cell line (derived from Cerebellum), The potential difference in Nicotine effect between human and mouse astrocytes, as well as astrocytes heterogenous sub-populations (region/functionality/morphological) should be discussed.
2. Discuss the extrapolation between study dosing and feeding regimen on potential in-vivo consumption
3. Propose directions/strategies to overcome the no specificity of A1/A2 markers, such as including additional markers, multi-omics, in-vivo analysis.
