# Supplementary material for: Nicotine alters cellular activity and mRNA expression of patterns of Astrocytes
Source: PLoS One. 2025 Jun 20;20(6):e0325529. doi: 10.1371/journal.pone.0325529 (PMC12180639; doi:10.1371/journal.pone.0325529)
Supplement: Attachment — Submitted filename: Comments-Li Feng.docx [file pone.0325529.s004.docx]

**Nicotine alters cellular activity and mRNA expression of patterns in vitro of murine**

**Astrocytes**

**Comments**

**By Li Feng**

**Jan 19, 2025**

**General Comments**
The manuscript addresses a significant public health concern by investigating the effects of nicotine on murine astrocytes, focusing on cellular activity and mRNA expression. The study is well-designed, employing appropriate methodologies to assess astrocyte responses to nicotine exposure. However, certain areas require further refinement to enhance clarity, rigor, and relevance to the broader scientific and public health community.

**Specific Comments on Sections**

1. **Introduction**
   - The introduction discusses the effects of nicotine during pregnancy, but it is unclear how this background connects to the study’s objectives. The authors should clarify:
     - Why is the focus on nicotine exposure during pregnancy relevant to astrocyte activity in this experimental model?
     - How does the current study design address the broader implications for pregnancy-related outcomes, if at all?
     - If the focus is more general (e.g., nicotine's effects on the central nervous system), consider rephrasing or narrowing the discussion for better alignment with the study's aim.
   - May need reference for “Discuss how findings from murine cells can be cautiously extrapolated to human physiology and potential limitations of this approach”.
2. **Materials and Methods**
   - **Cell Model**:
     - Why were murine astrocytic cells chosen instead of human astrocytic cells?
     - Discuss how findings from murine cells can be cautiously extrapolated to human physiology and potential limitations of this approach.
   - **Dosage Selection**:
     - Clarify the rationale for the chosen nicotine dosages at each experimental step.
     - Provide references or scientific justification for selecting the initial concentrations (0, 10, 25, 50, 100, 250, 500 ng/mL) and explain why the later experiments only used 0, 25, 50, and 100 ng/mL.
     - How do these concentrations relate to physiologically relevant exposures in humans (e.g., plasma nicotine levels in smokers or vapers)?
   - **Experimental Details**:
     - Ensure that dosages and time points are explicitly mentioned for each assay. This will help readers replicate and interpret the study.
3. **Results**
   - The description of findings is generally clear, but some terminology, such as "diminishing returns were observed," requires more explicit explanation for readers who may not be familiar with the concept.
   - Provide a brief interpretation of how observed changes in apoptosis and proliferation reflect potential mechanisms of nicotine's impact on astrocytes.
4. **Discussion**
   - Expand on the potential therapeutic implications of the findings (if possible):
     - How might astrocyte modulation contribute to strategies for addressing nicotine addiction and neuroinflammatory disorders?
     - Could targeting astrocytes play a role in smoking cessation interventions or reduce the neurological impact of nicotine exposure?
   - Broaden the discussion to include potential applications of the study's findings beyond pregnancy, such as the relevance of astrocyte responses to nicotine in adolescent brain development or chronic nicotine users if possible.
