# Supplementary material for: Nicotine alters cellular activity and mRNA expression of patterns of Astrocytes
Source: PLoS One. 2025 Jun 20;20(6):e0325529. doi: 10.1371/journal.pone.0325529 (PMC12180639; doi:10.1371/journal.pone.0325529)
Supplement: Attachment — Submitted filename: PONE-D-24-55873 - RR.docx [file pone.0325529.s006.docx]

**Response to Reviewers:**

***GENERAL COMMENTS***

***Reviewer I:***

- The manuscript addresses a significant public health concern by investigating the effects of nicotine on murine astrocytes, focusing on cellular activity and mRNA expression. The study is well-designed, employing appropriate methodologies to assess astrocyte responses to nicotine exposure. However, certain areas require further refinement to enhance clarity, rigor, and relevance to the broader scientific and public health community.

***Reviewer II:***

- The manuscript addresses an important topic. The study tests the effect of nicotine on murine astrocytes, focusing on cellular activity and mRNA expression. The results indicate that Nicotine impacts astrocyte activity and inflammatory signaling, suggesting its potential involvement in neuroinflammation.

***Specific Comments on Sections***

**ABSTRACT**

***Reviewer II:***

- The abstract as well as introduction should be modified to describe Tobacco/Nicotine consumption as a major global health concern/risk in general (in adults and in pregnancy), as nicotine teratological effect in astrocytic embryonic development was not specifically assessed in this paper. C8D1A cell line was used (Postnatal d8 astrocytic cerebellum).

*Response: We have updated the abstract and introduction to reflect this request. We note the cells were obtained as a cell line and maintained from a postnatal model. Most of our prior work on nicotine has been in the prenatal developmental sphere and we apologize for not taking a closer editing eye.*

**INTRODUCTION**

***Reviewer I:***

- The introduction discusses the effects of nicotine during pregnancy, but it is unclear how this background connects to the study’s objectives. The authors should clarify:
- Why is the focus on nicotine exposure during pregnancy relevant to astrocyte activity in this experimental model? *Response: As above it is not. We are interested in astrocyte reaction to any exposure including prenatal and have updated throughout to reflect this as the reviewer identified our approach was neither specific nor limited to a pregnancy exposure model.*
- How does the current study design address the broader implications for pregnancy-related outcomes, if at all? *Response: We have attempted to address this now in the discussion as we do have ongoing experiments assessing prenatal exposures on glial cells in vivo via a histological approach. As above, we apologize we were not more careful in our first submission.*
- If the focus is more general (e.g., nicotine's effects on the central nervous system), consider rephrasing or narrowing the discussion for better alignment with the study's aim. *Response: We hope our edits reflect our appreciation for the reviewer bringing this to our attention.*
- May need reference for “Discuss how findings from murine cells can be cautiously extrapolated to human physiology and potential limitations of this approach”. *Response: That is a fantastic point and not simply limited to murine to human comparison but also cell line vs primary cell approaches. We wanted a robust high throughput system for study and chose the murine astrocyte as it is an established cell line and with the fore knowledge, we were conducting prenatal exposure in an in vivo model we planned to study histologically for glial cell effects. We have added this to discussion, limitations, and future directions.*

***Reviewer II:***

- The introduction section should be expanded to include:
- Nicotine absorption, concentration in blood and brain, half-life (2 hours) etc. This would help understand the relevancy of selected doses and culture feeding regimen used in the study. *Response: Thank you for this comment. The selected feeding program was based on previous work by our own group on mesenchymal cell sensitivity to nicotine exposure, correlative cotinine concentrations for active nicotine use (30-300ng/ml) and our inclusive bioassays (FIGURE 1 and 2). We utilized both MTS and Apoptosis assay to define dose concentration and timepoints to be used for mRNA studies. Our range after considering IC50 and EC50 values led us to the 25-100ng/ml dose range that was used.*
- *Tobacco smoke comprises a complex mixture of hundreds of chemical compounds, many of which may potentiate the psychoactive properties of nicotine. Nonetheless, nicotine remains the principal agent underlying tobacco dependence, primarily through its capacity to reinforce drug-seeking and drug-taking behaviors. A key factor contributing to this dependence is nicotine’s relatively short plasma half-life of approximately two hours, indicating that half of the administered dose is metabolized and cleared from the body within that timeframe [10]. This rapid pharmacokinetic profile results in a transient duration of action, often compelling individuals to engage in frequent re-administration to sustain its psychoactive effects.*
- Summary of research exploring the effect of Nicotine on astrocytes should be expanded, include studies demonstrating the expression of nicotinic acetylcholine receptors (nAChRs) in astrocytes, and its downstream effects on astrocytic signaling pathways, morphological and functional changes etc. *Response: Thank you. We had previously edited this down for brevity but have included this now in discussion. We are relying primarily on published literature as in our own laboratory we have only conducted experiments with the alpha7 subunit for obvious reasons as it homodimerizes and is involved in addiction. We have expanded our discussion accordingly.*

**MATERIALS AND METHODS**

***Reviewer I:***

- **Cell Model:**
- Why were murine astrocytic cells chosen instead of human astrocytic cells? *Response: We are a preclinical teratology focused. Our next step is to use a translational in utero exposure model, so our preliminary experimentations were done with murine astrocyte cells.*
- Discuss how findings from murine cells can be cautiously extrapolated to human physiology and potential limitations of this approach. *Response: We have updated the discussion to address these concerns.*
  - ***Rationale for Using Murine Astrocytic Cells:***
    - *Murine astrocytic cells (C8D1A) were selected for this study due to their well-characterized, reproducible behavior in vitro and their widespread use as a model for studying astrocyte physiology and response to pharmacological agents. These cells provide a controlled and genetically stable platform for investigating mechanisms of cellular response, including proliferation, apoptosis, and gene expression. While human astrocytes offer species-specific insights, they present limitations such as donor variability, ethical constraints, and reduced scalability for high-throughput assays. Moreover, murine models remain highly relevant for translational research, as they are commonly used in in vivo studies of neuroinflammation, addiction, and neurodegeneration, facilitating continuity between in vitro and in vivo experimentation.*
- **Dosage Selection (ADDRESSED IN INTRO):**
- Clarify the rationale for the chosen nicotine dosages at each experimental step.
  - *Given the limited data on the specific effects of nicotine on astrocytes, we conducted targeted in vitro studies to determine whether astrocytes are responsive to nicotine and whether nicotine exposure alters their cell cycle in a dose- and time-dependent manner. Based on reported plasma nicotine concentrations following the consumption of a single cigarette (5–30 ng/mL; Benowitz & Jacob, 1994), we selected a range of nicotine concentrations (0, 10, 25, 50, 100, 250, and 500 ng/mL) to capture a spectrum of cellular responses. Subsequent analyses focused on concentrations (0, 25, 50, and 100 ng/mL) that elicited measurable cellular activity. In parallel, we assessed astrocytic mRNA expression profiles to test the hypothesis that nicotine exposure shifts the balance between neurotoxic/pro-inflammatory and neuroprotective/pro-reparative markers.*
- **Provide references or scientific justification for selecting the initial concentrations** (0, 10, 25, 50, 100, 250, 500 ng/mL) and explain why the later experiments only used 0, 25, 50, and 100 ng/mL. *Response: These doses allowed for maximal effect without unnecessary toxicity.*
  - *After preliminary experimentation, two concentrations of nicotine, namely 25ng/ml and 50ng/ml, were selected based on their significance as determined by cell viability assays. Cell viability, indicative of proliferation, was evaluated using the colorimetric MTS assay. Apoptosis was assessed using the Apo-ONE Caspase-3/7 fluorescence assay. The results from cell viability assays led to the determination of 25ng/ml and 50ng/ml being those concentrations of interest. The 100ng/ml nicotine concentration was added to gauge effects of an exorbitant exposure of nicotine. We also decided to explore these concentrations at 24-, 48-, and 72-hour time points.*
  - How do these **concentrations relate to physiologically relevant exposures in humans (e.g., plasma nicotine levels in smokers or vapers)**? *RESPONSE: Literature suggests 30-300 ng/ml is indicative of active nicotine use.*
  - *Active smokers typically consume between 10 and 100 mg of nicotine per day, while individuals using alternative nicotine delivery systems may be exposed to an even broader range of nicotine levels (Benowitz 1984; L Benowitz, et al 1988)*
  - **Experimental Details:**
  - **Ensure that dosages and time points are explicitly mentioned for each assay**. This will help readers replicate and interpret the study. *Response: This has been updated throughout.*

***Reviewer II:***

- **Material and Methods:**
- **Please include media change regimen** (e.g. daily? Every other day? Etc.) – might affect results interpretation (Acute exposure vs. chronic). *All regimens were acute and only necessitated one feed as indicated by the initial dose schema gating of the MTS and Caspase assays (Figures 1 and 2)*
- **Include Nicotine source and Cat number.**
  - *Sigma Life Science (−)-Nicotine ≥99% (GC), liquid N3876-100ml CAS Number 54-11-5 (IN TEXT: Sigma Aldrich, St. Louis, MO, USA, N3876)*

**RESULTS**

***Reviewer I:***

- The description of findings is generally clear, but some terminology, such as "diminishing returns were observed," requires more explicit explanation for readers who may not be familiar with the concept. *Response: We have updated our language for consistency.*
  - *C8D1A cell viability following nicotine exposure was assessed using the MTS assay at 12, 18, 24, and 48 hours. Early time points (12 and 18 hours) revealed a modest reduction in cell viability across most nicotine concentrations, with a slight rebound observed at 100 ng/mL at 12 hours and a similar trend at 18 hours following 50 ng/mL treatment. These findings suggest an initial sensitivity of astrocytes to nicotine. By 24 and 48 hours, nicotine exposure exhibited a stimulatory effect on cell proliferation across all tested concentrations. However, this proliferative response plateaued at concentrations above 50 ng/mL, indicating that increasing nicotine concentration beyond 50 ng/mL did not result in proportionally greater increases in cell proliferation—the beneficial effect of nicotine started to level off, or even decrease, despite higher doses (Figure 1)*
- Provide a brief interpretation of how observed changes in apoptosis and proliferation reflect potential mechanisms of nicotine's impact on astrocytes. *Response: Although we did interpret the time course in this vein the initial experiments were simply conducted as a pharmacological bioassay to define critical concentrations to be tested for mRNA marker studies focused on astrocyte polarization.*

***Reviewer II:***

- **MTS Assay:**
- The assay relies on cellular metabolic activity, which may not always directly correlate with cell number, if feasible, please correlate with number of counted cells (e.g., nucleocounter/Hemocytometer, ICF, DAPI Count). If not applicable, address this issue in the discussion section. *Response: We have added this to the limitations, and although we agree MTS is still a standard in the field of experimental pharmacology and drug development to define IC25/50/75/90 and EC25/50/75/90 values, etc. We used these tools is a similar fashion to identify critical concentration schema for polarization studies.*
- *MTS assays were utilized in our cell culture studies to obtain a more accurate and functionally relevant assessment of cell viability. Unlike direct cell counting methods, which merely quantify total cell numbers, the MTS assay provides insight into cellular metabolic activity. Aside from determining initial seeding densities, cell counts were not employed, as they do not adequately reflect the metabolic status or viability of the cultured cells.*
- **Figure 1.** Where applicable please add statistical significance asterisks (e.g. in comparison with non-treated-NT arm (0ng/ml) at same time point). The Y axis should be modified to represent Cell Viability (measured by OD).  *Response: We would be happy to revisit this if considered necessary given our above explanation of how we utilized our MTS and apoptosis information.*
- The reduction in cell activity/cell number or metabolic activity in un-treated arm after 24 & 48 hours compared to 12&18hr should be explained. *Response: This is likely an artifact of cell culture on plastic, cell loss is often observed in the first 24-48 hours after plating which is why control 0ng/ml group is so critical for interpretation.*
  - **Figure 2.** Where applicable statical significance (asterisks) compared to NT should be added, Y-axis (apoptosis – measured by RFU – add abbreviation (Relative Fluorescence Units). *Response: We would be happy to revisit this if considered necessary given our above explanation of how we utilized our MTS and apoptosis information.*
  - **Figure 3 & 4:** The peak in mRNA expression (in both A1/A2 markers graphs) occurs after 48 hr. (higher fold change – pls note difference in scaling), should be discussed/explained. *Response: As we are not comparing between timepoints we did not use the same y axis scale. We would be happy to revisit. However, we interpret the highest values changes as being the critical signaling axis peak after acute exposure. We have added this to the discussion.*

**DISCUSSION**

***Reviewer I:***

- Expand on the potential therapeutic implications of the findings (if possible):
- How might astrocyte modulation contribute to strategies for addressing nicotine addiction and neuroinflammatory disorders? *Response: We would like to thank the reviewer for prompting our expansion of the discussion. Astrocytes have been linked to drug seeking behaviors and transcriptional changes are crucial to interpreting biological responses to nicotine we do believe that the astrocyte may prove a useful target in the monitoring of inflammatory mechanistic processes related to spectrum of nicotine related health problems.*
- Could targeting astrocytes play a role in smoking cessation interventions or reduce the neurological impact of nicotine exposure? *Response: As astrocytes play a critical role in nicotine use and cessation relapse, we do believe that targeted therapies that alter extracellular levels of certain neurotransmitters (i.e. dopamine) associated with astrocyte activity may prove to be a useful therapeutic tool.*
- Broaden the discussion to include potential applications of the study's findings beyond pregnancy, such as the relevance of astrocyte responses to nicotine in adolescent brain development or chronic nicotine users if possible. *Response: We agree our focus was previously too heavily reliant on interpretations of prenatal exposures. We have updated throughout.*

***Reviewer II:***

- The effect of Nicotine was assessed only in one murine cell line (derived from Cerebellum), The potential difference in Nicotine effect between human and mouse astrocytes, as well as astrocytes heterogenous sub-populations (region/functionality/morphological) should be discussed. Response: Agreed. This is similar to a previously addressed comment above.
  - Discuss the extrapolation between study dosing and feeding regimen on potential in-vivo consumption. *Response: Agreed. This is similar to a previously addressed comment above.*
  - Propose directions/strategies to overcome the no specificity of A1/A2 markers, such as including additional markers, multi-omics, in-vivo analysis. *Response: Our future work is moving in multiple directions. One is to expand our markers utilizing a next generation sequencing approach to garner a greater understanding of the acute effects of nicotine on astrocyte cellular signaling. Our other major direction that is nearing completion of a preliminary analysis is in utero exposures on the glial cellularity and activity when assessed histologically in the early post-natal period (prolonged effects after birth)*
